# Ectasie cornéenne suite à une brûlure thermique

**DOI:** 10.11604/pamj.2016.25.257.11087

**Published:** 2016-12-21

**Authors:** Mehdi Khmamouche, Zerrouk Rachid

**Affiliations:** 1 Service d’Ophtalmologie, Hôpital Militaire d’Instruction Mohamed V, Rabat, Maroc

**Keywords:** Ectasie cornéenne, kératoplastie, brölure, Corneal ectasia, keratoplasty, burn

## Image en médecine

L’ectasie cornéenne est une complication rare qui met en jeu la fonction visuelle. Son début parfois insidieux et retardé dans le temps, son caractère non réversible et son pronostic péjoratif (elle peut motiver l’indication d’une greffe de cornée) sont autant de caractéristiques qui en font une complication redoutée, et lui confèrent une dimension adverse voire pernicieuse. La brûlure thermique peut être en cause. Nous rapportons le cas d’un patient âgé de 65 ans, sans antécédents particuliers, qui a présenté une ectasie cornéenne de l’œil droit suite à une brûlure thermique par l’huile de table depuis une année. L ’acuité visuelle était limitée à une perception lumineuse positive. L’examen du segment antérieur à la lampe à fente a objectivé une ectasie cornéenne avec amincissement central important, des opacités stromales et une distension limbique sans appel vasculaire. L’échographie oculaire n’a pas montré d’anomalies du segment postérieur associée. Le patient a bénéficié d’une kératoplastie transfixiante ayant donné de bons résultats esthétiques (réfection du segment antérieur) et une légère récupération visuelle (acuité visuelle: mouvements des doigts).

**Figure 1:**
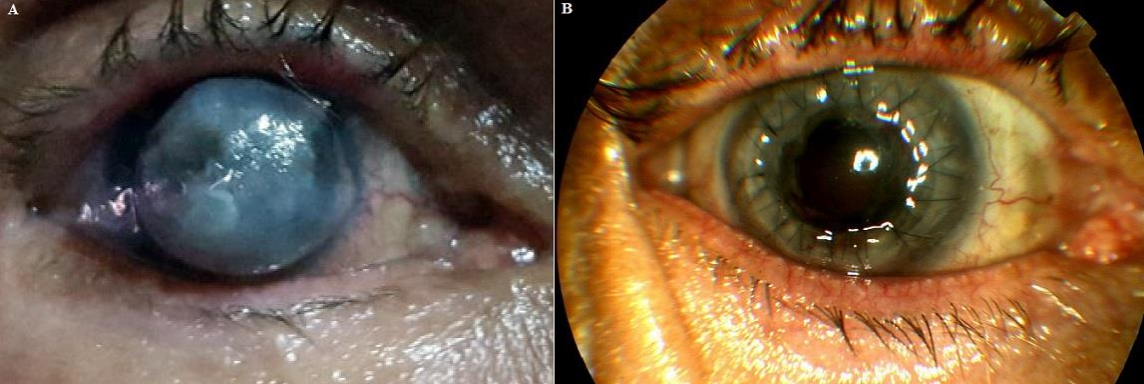
A) ectasie cornéenne; B) image après kératoplastie

